# Diffusion of Charged Species in Liquids

**DOI:** 10.1038/srep35211

**Published:** 2016-11-04

**Authors:** J. A. del Río, S. Whitaker

**Affiliations:** 1 Instituto de Energías Renovables, Universidad Nacional Autonoma de Mexico, A.P. 34, 62580 Temixco, Mor. Mexico; 2 Department of Chemical Engineering and Material Science, University of California at Davis, Davis, CA 95616, USA

## Abstract

In this study the laws of mechanics for multi-component systems are used to develop a theory for the diffusion of ions in the presence of an electrostatic field. The analysis begins with the governing equation for the *species velocity* and it leads to the governing equation for the *species diffusion velocity*. Simplification of this latter result provides a momentum equation containing three dominant forces: (a) the gradient of the partial pressure, (b) the electrostatic force, and (c) the diffusive drag force that is a central feature of the Maxwell-Stefan equations. For ideal gas mixtures we derive the classic Nernst-Planck equation. For liquid-phase diffusion we encounter a situation in which the Nernst-Planck contribution to diffusion differs by several orders of magnitude from that obtained for ideal gases.

The study of ion transport in fluids is an important topic with a wide range of applications. Some classic examples are batteries, fuel cells, electroplating, and protection of metal structures against corrosion^1^. In addition to the traditional battery, the flow battery or rechargeable fuel cell^2^,^3^ represents an important new technology involving the transport of ions. Ion exchange membranes have a wide range of applications^4^, and the underlying theory has been a matter of concern for several decades^5^. Other examples of complex electro-chemical systems are the transport of charged particles in ion channels^6^,^7^,^8^, in protein channels^9^, and during the primordial conversion of light to metabolic energy^10^. Often upscaling is necessary for a complete analysis of the transport of electrolytes in charged pores^11^,^12^.

Much of ion transport occurs at the nano-scale^13^, and most of the studies use the Nernst-Planck equation^14^,^15^,^16^ to describe this type of phenomena. However, there are molecular dynamic simulations indicating that the Nernst-Planck equation does not always provide a complete description^17^, and there are experimental studies that lead to the same conclusion^18^. The need to analyze the limits of the Nernst-Planck contribution to diffusion has been emphasized^19^ in an exploration of the transport of divalent ions in ionic channels.

The authors of this paper have not found a derivation of the Nernst-Planck equation that *does not* make use of the ideal gas assumption. To be precise we note that ideal gas behavior for mixtures is based on Dalton’s laws (see page 114 in ref. 20) that we list as


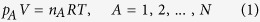



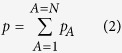



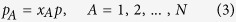


Some care must be taken with the interpretation of Eq. 2 since it applies to all Stokesian fluids and is therefore not limited to ideal gas mixtures (see Appendix B in ref. 21). Caution must also be used with the interpretation of Eq. 3 which is applicable to all ideal fluid mixtures. For example, Eq. 3 should provide reliable results for a liquid mixture of hexane and heptane, but it should not for a mixture of ethanol and water.

Here it is important to emphasize that Eq. 1 can only describe a fluid composed of non-interacting particles. To provide a reliable framework in which a fluid is considered to be composed of interacting particles — as in the case of a liquid — it is necessary to understand the process of Nernst-Planck diffusion in a fluid different from an ideal gas. This is the problem under consideration in this paper.

In this work we analyze the ion transport process from a fundamental point of view using an axiomatic mechanical perspective. Our analysis is for ideal *fluid* mixtures, and from the analysis we find that the classic Nernst-Planck relation is only valid for ideal *gas* mixtures. For ideal *liquid* mixtures we find an alternative expression in which the form is similar to that for ideal *gas* mixtures, but significant other terms appear in the final result.

In the following section we present a detailed analysis based on axiomatic statements for the mechanics of multi-component systems. Our objective is a search for clarity and rigor in terms of a diffusion equation that is applicable to both *gas* and *liquid* mixtures, i.e., applicable to *fluid* mixtures. We first derive a general equation for the species velocity, and from this we extract a general equation for the species *diffusion* velocity. This equation is the origin of Fick’s law of diffusion; however, the derivation of Fick’s law from the governing equation for the species diffusion velocity is complex. We begin our study of the general equation for the species diffusion velocity with a series of plausible restrictions that lead to a special form of the governing equation for the diffusion velocity. This provides a transport equation for the diffusion of charged molecules in *fluids*, and this general form can be used to clearly establish that the classic Nernst-Planck equation is only valid for ideal *gas* mixtures.

## Analysis

**Figure 1:**
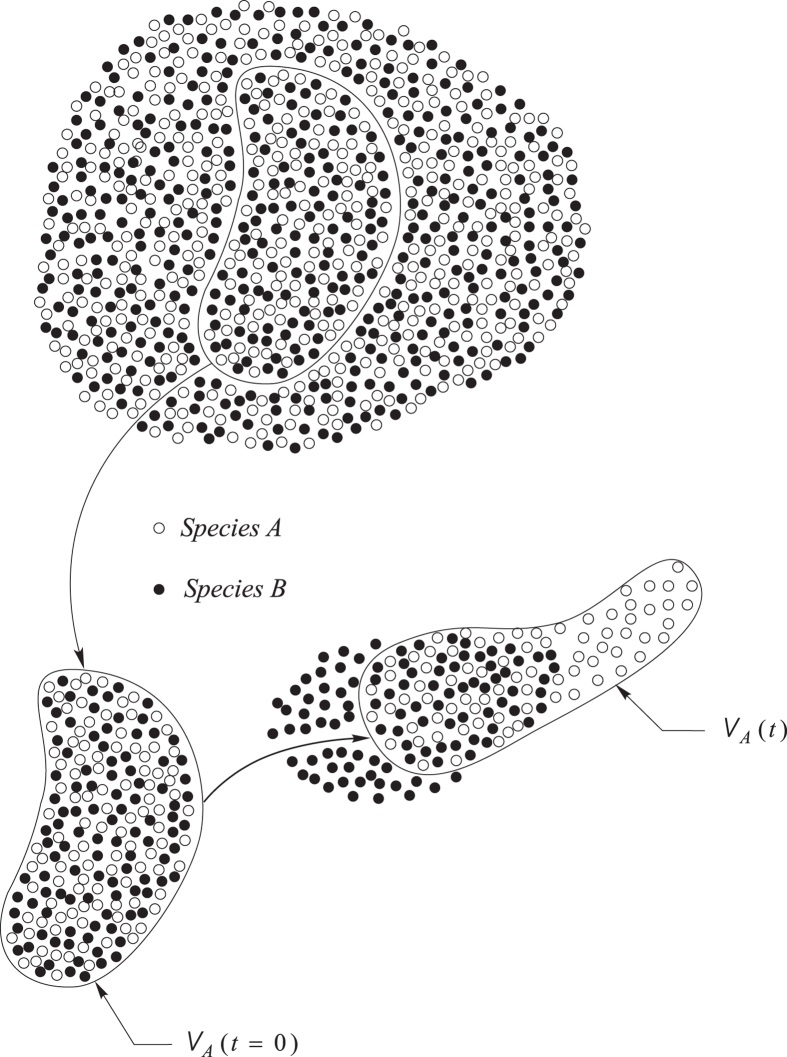
Species *A* Body.

The analysis in this study is presented in terms of axiomatic statements concerning the species *A* body illustrated in Fig. 1. There we have used *V*_*A*_(*t*) to identify the volume occupied by the species *A* body, and we have illustrated only the presence of one other species, species *B*; however, one should image the presence of many other species. We begin with a review of the axioms for mass and mechanics of multi-component systems, and then move on to explore the dominant terms in the species momentum equation. If one accepts the simplification that the electric field represents a specified force, the motion of both charged and uncharged species can be treated in terms of the axioms for mass and momentum that are given in the following paragraphs. It is crucial to understand that the following development is applicable to both gases and liquids.

### Mass

We begin our study with the two axioms for mass given by


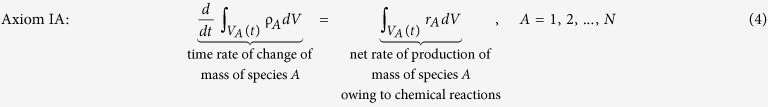



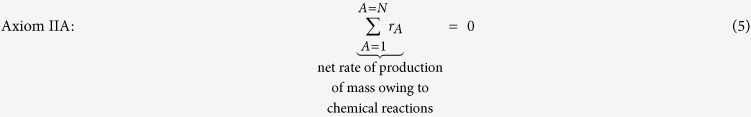


The volume of the species *A* body is represented by *V*_*A*_(*t*) and the speed of displacement of the surface of this volume is **v**_*A*_·**n**. Axiom I A can be used to derive the species *A* continuity equation by using the transport theorem and the divergence theorem (see Sec. 1.3.2 in ref. 22). This leads to


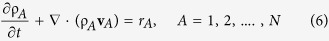


The total continuity equation is obtained by summing Eq. 6 over all *N* species and imposing Eq. 5 in order to obtain (see page 83 in ref. 23)


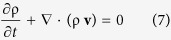


Here the total density, ρ, and the total mass flux, ρ**v**, are defined by


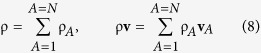


The mass fraction of species *A* is defined according to


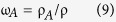


and use of this definition with Eq. 8 provides a constraint for the mass fractions and a representation for the mass average velocity. These two results take the form


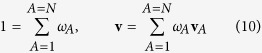


The objective of this study is the development of an expression for the diffusive flux of species *A*. This requires that we decompose the species velocity according to


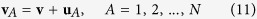


in which **u**_*A*_ is the *mass diffusion velocity*. This decomposition allows us to express Eq. 6 in terms of convective and diffusive fluxes according to (see Eq. 19.1–7 in ref. 23)


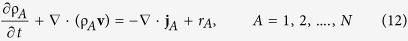


Here we note that the mass diffusive flux is defined by


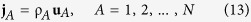


and this flux is constrained by


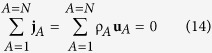


Given these forms of the species continuity equation and the total continuity equation, we are ready to move on to the analysis of the mechanics of multi-component fluids.

### Mechanics

The first of the four axioms associated with the species *A* body is the *linear momentum principle* given by (see page 85 in ref. 24)


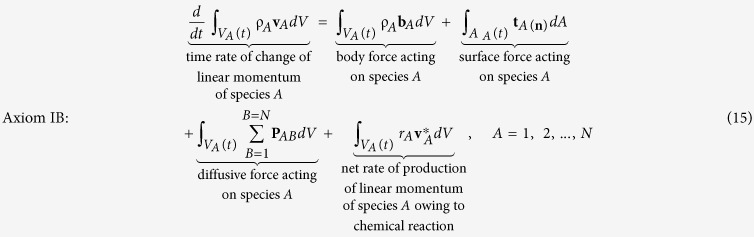


Here the species stress vector, **t**_*A*(**n**)_, represents the force that the *surroundings* (into which **n** is directed) exert on species *A within* the species *A* body. We have used **P**_*AB*_ to represent the *diffusive force* exerted by species *B* on species *A* and it is understood that


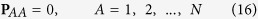


In the last term in Eq. 15 we have indicated the possibility that species *A* entering or leaving the species *A* body owing to chemical reaction may have a velocity 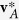 different than the continuum velocity **v**_*A*_.

The second axiom is the *angular momentum principle* that takes the form


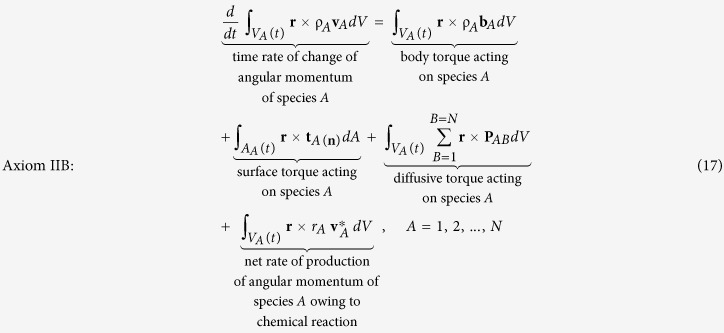


Here **r** represents the position vector relative to a fixed point in an inertial frame. Truesdell (see page 85 in ref. 24) presents a more general version of Axiom II B in which a *growth of rotational momentum* is included, and Aris (see Sec. 5.13 in ref. 25) considers an analogous effect for polar fluids.

The third axiom requires that there be no net diffusive force in the total momentum equation (see page 133 in ref. 26) and we express this idea as


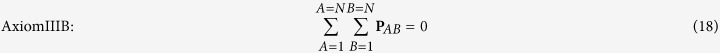


In terms of chemical reactions, Hirschfelder *et al*. (see page 497 in ref. 27) point out that “even in a collision which produces a chemical reaction, mass, momentum and energy are conserved”. The continuum version of this idea for linear momentum gives rise to the fourth axiom that we express as


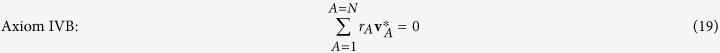


Returning to Eq. 15 we note that Axiom I B can be used to develop Cauchy’s lemma given by (see Section 203 in ref. 28)


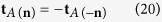


in which **t**_*A*(−**n**)_ represents the force that species *A* exerts *on the surroundings*. Cauchy’s fundamental theorem for the species stress vector is given by (see Lecture 5 in ref. 24).


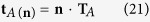


and one can use this result in Eq. 15 to obtain the following

Species *A* Momentum Equation:


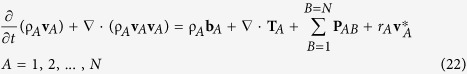


At this point we can use the continuity equation given by Eq. 6 to obtain


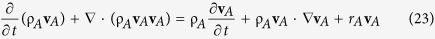


and this allows us to express Eq. 22 in the form


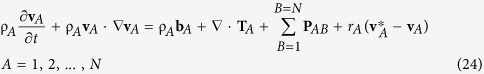


Both Eq. 22 and Eq. 24 can be found in the literature; however, locating them requires some effort because of the lack of a uniform nomenclature (see Sec. 1.2 in ref. 21). Chapman and Cowling (see page 135 in ref. 29) obtained Eq. 24 for dilute gases with *r*_*A*_ = 0 by means of a generalized equation of change. In addition, this result was presented by Truesdell and Toupin (see Eq. 215.2 in ref. 28) and by Truesdell (see Eq. 22 in ref. 30). Bearman and Kirkwood (see Eq. 4.20 in ref. 31) presented Eq. 22 for the case of *r*_*A*_ = 0 as did Curtiss and Bird (see Eq. A2 in ref. 32). Datta and Vilekar (see Eq. 2 in ref. 33) have derived Eq. 22 beginning with Eq. 15, and Jou *et al*. (see Eq. 1.21 in ref. 34) present Eq. 22 with the last two terms identified as “…contributions due to the interaction forces and the exchange of momentum between various components…” Most recently the developments leading to Eqs 22 and 24 have been summarized by Whitaker (see ref. 35).

The analysis of Axiom II B is rather long; however, after some algebra one finds that Eq. 17 leads to the symmetry of the species stress tensor indicated by (see Lecture 5 in ref. 24)


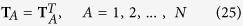


This is identical in form to Cauchy’s second law of motion (see page 187 in ref. 36). At this point it seems clear that there is general agreement concerning the form of the species linear momentum equation that represents the governing equation for the species velocity, **v**_*A*_. In the study of diffusion processes, it is the diffusion velocity, **u**_*A*_, that is important, and in the following paragraphs we develop the governing equation for this velocity.

## Diffusion Velocity

The total linear momentum equation is obtained by summing Eq. 22 over all species and imposing the axioms given by Eqs 18 and 19. This leads to


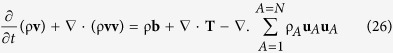


in which the total stress tensor and the total body force are defined by


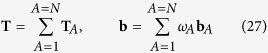


At this point we recall that a Stokesian fluid (see Sec. 59 of ref. 37, see Sec. 5.21 of ref. 25, see Secs. 3.3 and 3.4 of ref. 38, see Sec. 2.3.2 of ref. 22) can be described by:


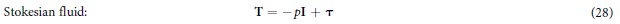


Here **τ** is the *viscous stress tensor*, and *p* is the *local thermodynamic pressure* given by


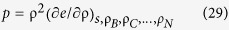


in which *e* is the internal energy per unit mass. To relate this expression for the pressure to that given by Slattery (see page 444 of ref. 22) and to that given by Callen (see Eq. 2.4 of ref. 39), one makes use of the following


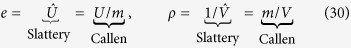


Here *m* represents the mass of the closed system preferred by Callen^39^. Use of Eq. 28 with Eq. 26 allows us to express the *total momentum equation* in the form


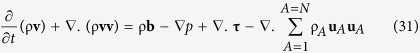


Returning to the *species momentum equation* given by Eq. 22, we propose that the species stress tensor can be represented by


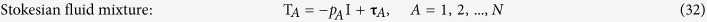


Here *p*_*A*_ is the partial pressure defined by (see Chapter 10 of ref. 40)


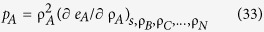


in which *e*_*A*_ is the internal energy of species *A* per unit mass of species *A*. The classic relation between the partial pressures and the total pressure is given by


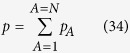


and this *requires* that


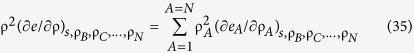


The proof of this result is rather long and is given by Whitaker (see Appendix B of ref. 21). We can use Eq. 27 through Eq. 34 to conclude that


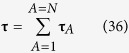


It is important to recognize that the application of Eqs 29 and 33 requires an assumption of *local thermodynamic equilibrium* (see Sec. 3.4 of ref. 38), and this should be satisfactory for many, but not all, mass transfer processes. The use of Eq. 32 in Eq. 22 leads to the following form of the species *A* momentum equation


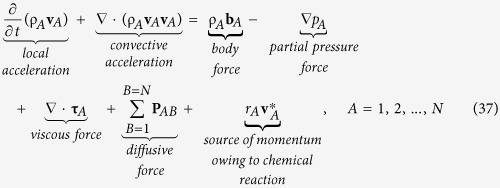


Here one should note that the diffusive force is constrained by Eq. 16.

At this point it will be convenient to use Eq. 7 with Eq. 26 to obtain the following form of the *total* momentum equation


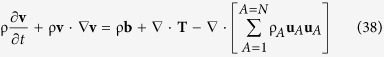


The analogous form of the *species* momentum equation is given by Eq. 24 and repeated here as


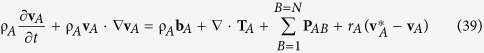


At this point we wish to use Eqs 38 and 39 to derive the governing equation for the mass diffusion velocity, **u**_*A*_. We begin by multiplying Eq. 38 by the mass fraction ω_*A*_ leading to


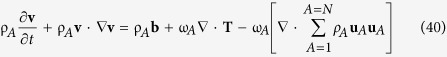


and we subtract this result from Eq. 39 to obtain the desired *governing differential equation* for the mass diffusion velocity, **u**_*A*_, given by


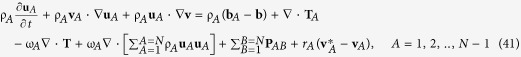


This governing equation for **u**_*A*_ is the origin of Fick’s law (see Eq. 1 of ref. 41; see page 45 of ref. 42); however, some simplifications are required in order to achieve that classic result. To be clear about this point, we note that Fick’s law for a binary system is given by (see Table 17.8-2 of ref. 23)


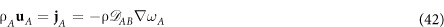


and the path between Eq. 41 and Eq. 42 is rather long (see Eq. 110 of ref. 21).

We now make use of Eqs 28 and 32 with Eq. 41 to obtain the following form of the governing equation for the species *A* mass diffusion velocity


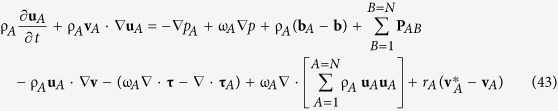


Plausible restrictions associated with this governing equation for **u**_*A*_ are given by (see Sec. 1.2 in ref. 21) and are listed here as


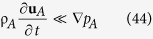



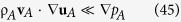



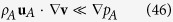



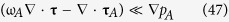



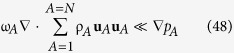



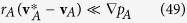


An effort to justify these *restrictions* is given by Whitaker (see Secs. 2.6 and 2.7 in ref. 43); however, a rigorous analysis leading to *constraints* is still unavailable. Equation 44 indicates that the governing equation for **u**_*A*_ is *quasi-steady*; Eq. 45 indicates that *diffusive inertial effects* are negligible, Eq. 46 indicates that the influence of *mass average rotational flow* is negligible, Eq. 47 indicates that *viscous effects* are negligible, Eq. 48 indicates that *diffusive stresses* are negligible, and Eq. 49 indicates that the effects of *homogeneous chemical reactions* are negligible. When the restrictions given by Eqs 44 through 49, 76, 76, 76, 76, 76 are imposed, the governing equation for the mass diffusion velocity takes the form


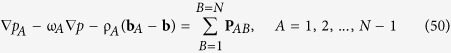


Curtiss & Bird (see Eq. 7.6 in ref. 44) represent the left hand side of Eq. 50 by *cRT***d**_*A*_ and refer to it as the *generalized driving force for diffusion*. Truesdell (see Eq. 7 in ref. 30) represents the left hand side of Eq. 50 by *p***d**_*A*_ and cites^27^ as the source. To illustrate the connection with^27^, we need Eq. 3 repeated here as


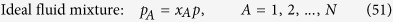


Use of this representation for the partial pressure indicates that our analysis is restricted to *ideal fluid mixtures* and it allows us to express Eq. 50 in the form


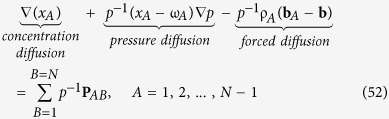


The left hand side of this result is *identical* to Enskog’s perturbation solution^45^ (see Eq. 7.3–27 in ref. 27). This indicates that the simplifications represented by Eqs 44 through 49 are c*onsistent with* the perturbation analysis^45^. It is important to keep in mind that Eq. 50 represents a special form of the governing equation for the diffusion velocity, **u**_*A*_, subject to the restrictions indicated by Eqs 44 through 49, 76, 76, 76, 76, 76. In addition, this equation for the diffusion velocity is restricted by the thermodynamic results indicated by Eqs 29 and 33. Finally, we remind the reader that Eq. 50 is a continuum result that is not restricted to either gases or liquids but is restricted to Stokesian fluid mixtures.

We begin our analysis of Eq. 52 by neglecting the pressure diffusion term. This represents a satisfactory simplification for many diffusion processes; however, it is not acceptable for centrifugation processes (see page 776 in ref. 23; see page 452 in ref. 42). Given this simplification, Eq. 52 takes the form


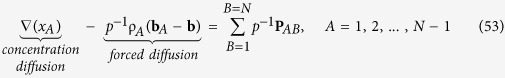


and we are ready to analyze the term on the right hand side.

## Diffusive Force

From dilute gas kinetic theory we know that **P**_*AB*|*gas*_ is given by


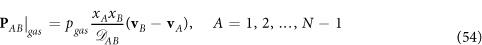


This result can be obtained by comparing Eq. 52 of this work with Eqs 7.3–27 and 7.4–48 of ref. 27 and neglecting the effect of thermal diffusion. At this point we need a representation for **P**_*AB*_|_*liq*_, and one possibility is to use Eq. 54 as a *model*. This leads to


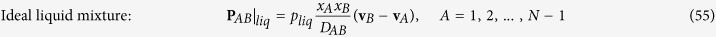


where it is understood that all the terms on the right side are associated with a liquid mixture. It should be clear that Eq. 55 represents an *empirical expression* for the diffusive force, **P**_*AB*_|_*liq*_, and that *D*_*AB*_ represents an *empirical coefficient* to be determined by experiment. We can *generalize* Eqs 54 and 55 to the single form


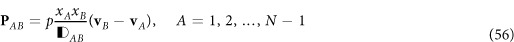


in which the diffusivity 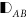 is given by


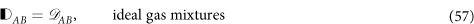



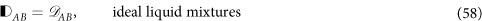


Use of Eq. 56 in Eq. 53 leads to


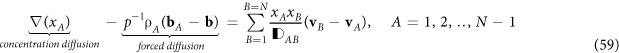


In Appendix A we show that for dilute solutions we obtain the result given by


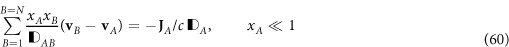


Here **J**_*A*_ represents the mixed-mode diffusive flux (see page 537, Eq. H in ref. 23, see Sec. 4, Eq. 90 in ref. 21) defined according to


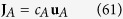


and 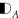 is a mixture diffusion coefficient given by


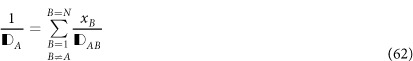


We refer to **J**_*A*_ as the mixed-mode diffusive flux since it is made up of the *molar* concentration, *c*_*A*_, and the *mass* diffusion velocity, **u**_*A*_.

Use of Eq. 60 in Eq. 59 allows us to express the mixed-mode diffusive flux according to


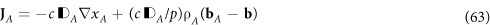


At this point we make use of the analysis given in Appendix B where we demonstrate the well-known result given by (see page 776 in ref. 23)


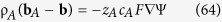


Here *z*_*A*_*F* represents the *charge per mole* of species *A* in which *z*_*A*_ is the valence (i.e., −2 for 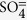 and +1 for H_3_O^+^) and *F* is the conversion factor known as Faraday’s constant. It is important to note that Eq. 64 is subject to the approximation of electro-neutrality (see page 454 in ref. 42; see ref. 46) and this simplification will not be valid near charged surfaces^11^. Use of Eq. 64 in Eq. 63 leads to the following result


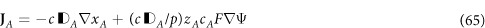


At this point we impose the dilute solution condition given by


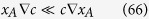


so that Eq. 65 takes the form


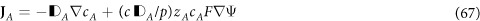


In order to represent the second term on the right hand side of Eq. 67 in terms of what is usually called Nernst-Planck diffusion, we introduce the product *RT* and arrange that term to obtain the following result


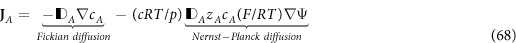


For *ideal gas mixtures* we have *cRT*/*p* = 1 and the diffusivity takes the form 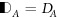 as indicated by Eq. 57, Eq. A10 and Eq. A13. Under these circumstances Eq. 68 simplifies to the classic Nernst-Planck equation (see page 149 in ref. 47, see Eq. 3 in ref. 11, see Eq. 11. in ref. 42, see page 1844 in ref. 48) given by


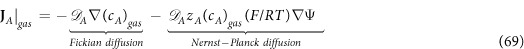


For liquid mixtures we have *cRT*/*p* = *c*_*liq*_*RT*/*p*_*liq*_ and the diffusivity takes the form 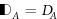 as indicated by Eq. 58, Eq. A10, and Eq. A16. Under these circumstances Eq. 68 takes the form


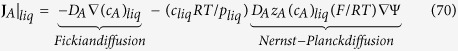


At this point it is important to realize that *c*_*liq*_*RT*/*p*_*liq*_ ≈ 1200 and there is *no simplification* of Eq. 68 to the form given by Eq. 69 for *liquid phase diffusion*. The development based on Eqs 54 and 55 is not the only route to Eqs 69 and 70, and another approach making use of the relation *p*_*ga*_ = *c*_*gas*_*RT* is given in Appendix C.

The analysis presented here indicates that the *classic result* for dilute solution diffusion of ions in an electric field is only valid for *ideal gas mixtures*. When confronting this situation one must keep in mind that the results given in Appendix A for the third term in Eq. 53 would appear to be universally accepted (see page 47 in ref. 49, see page 19 in ref. 47, see page 448 in ref. 42). In addition, the result given in Appendix B for the second term in Eq. 53 would also appear to be universally accepted (see page 279 in ref. 49, see page 454 in ref. 42, see page 776 in ref. 23).

## Conclusions

In this paper we have derived an ion transport equation for *ideal fluid mixtures*, and we have shown that the *classic* Nernst-Planck equation applies only to *ideal gas mixtures*. For *ideal liquid mixtures*, the laws of mechanics, the laws of electrostatics, and the thermodynamic representation for the partial pressure all lead to a new result. Non-ideal gases and liquids can be analyzed using the approach presented in this paper; however, that subject has not been explored in this work^50^,^51^.

**Nomenclature**

**b**_*A*_ = body force per unit mass exerted on species *A*, N/kg

**b** = 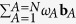, body force per unit mass exerted on the mixture, N/kg

*c*_*A*_ = molar concentration of species *A*, mol/m^3^

*c* = total molar concentration, mol/m^3^


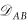
 = 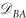, ideal gas binary diffusion coefficient for species *A* and *B*, m^2^/s


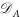
 = ideal gas mixture diffusivity for species *A*, m^2^/s

*D*_*AB*_ = *D*_*BA*_, dilute solution liquid-phase binary diffusion coefficient for species *A* and *B*, m^2^/s

*D*_*A*_ = dilute solution mixture diffusivity for species *A*, m^2^/s

**E** = electric field, V/m

*F* = Faraday’s constant, 9.652 × 10^4^ coulombs/mol

**g** = gravitational body force per unit mass, N/kg

**l** = unit tensor

**J**_*A*_ = *c*_*A*_**u**_*A*_, mixed-mode diffusive flux of species *A*, mol/ m^2^s

**n** = unit normal vector

*N* = total number of molecular species

**P**_*AB*_ = diffusive force per unit volume exerted by species *B* on species *A*, N/m^3^

*p*_*A*_ = partial pressure of species *A*, N/m^2^

*p* = 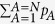, total pressure, N/m^2^

*r*_*A*_ = net mass rate of production of species *A* owing to homogeneous reactions, kg/m^3^s

*R* = gas constant, cal/mol K50

*t* = time, s


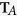
 = stress tensor for species *A*, N/m^2^


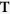
 = total stress tensor, N/m^2^

*T* = temperature, K

**u**_*A*_ = **v**_*A*_ − **v**, mass diffusion velocity, m/s

**v**_*A*_ = velocity of species *A*, m/s

**v** = 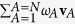, mass average velocity, m/s

*x*_*A*_ = *c*_*A*_/*c*, mole fraction of species *A*

*z*_*A*_ = valence (±) for ionic species *A*

Greek Letters

ρ_*A*_ = mass density of species *A*, kg/m^3^

ρ = total mass density, kg/m^3^

**τ** = viscous stress tensor, N/m^2^

**τ**_*A*_ = viscous stress tensor for species *A*, N/m^2^

ω_*A*_ = ρ_*A*_/ρ, mass fraction of species *A*

Ψ = electric potential function, V

## Additional Information

**How to cite this article**: del Río, J. A. and Whitaker, S. Diffusion of Charged Species in Liquids. *Sci. Rep*. **6**, 35211; doi: 10.1038/srep35211 (2016).

## Supplementary Material

Supplementary Appendix A

Supplementary Appendix B

Supplementary Appendix C
