# Supplementary material for: Diffusion of Charged Species in Liquids
Source: Sci Rep. 2016 Nov 4;6:35211. doi: 10.1038/srep35211 (PMC5566185; doi:10.1038/srep35211)
Supplement: Supplementary Appendix A [file srep35211-s1.pdf]

Diffusion of Charged Species in Liquids

J. A. del Río[1]* and S. Whitaker[2]

1.Instituto de Energías Renovables, Universidad Nacional Autonoma de Mexico, A.P. 34, 62580 Temixco, Mor. Mexico

2. Department of Chemical Engineering and Material Science, University of California at Davis, Davis, CA 95616, USA

* Correspondence
Dr. J.A. del Río,
Email: arp@ier.unam.mx

September 5, 2016

Appendix A: Diffusive force

We begin this analysis with Eq. 56 repeated here as

$$\mathbf{P}_{AB} = p \frac{x_A x_B}{\mathscr{D}_{AB}} (\mathbf{v}_B - \mathbf{v}_A), \quad A = 1,2,...,N-1 \tag{A1}$$

and note that this result takes the special forms indicated by Eqs. 54 and 55 for ideal gas and ideal liquid mixtures. The sum of this result is given by

$$\sum_{B=1}^{B=N} \mathbf{P}_{AB} = p \sum_{B=1}^{B=N} \frac{x_A x_B}{\mathscr{D}_{AB}} (\mathbf{v}_B - \mathbf{v}_A), \quad A = 1,2,...,N-1 \tag{A2}$$

and representation in terms of the diffusion velocity (see Eq. 11) leads to

$$\sum_{B=1}^{B=N} \mathbf{P}_{AB} = p \sum_{B=1}^{B=N} \frac{x_A x_B}{\mathscr{D}_{AB}} (\mathbf{u}_B - \mathbf{u}_A), \quad A = 1,2,...,N-1 \tag{A3}$$

The right hand side of this result can be expanded to provide

$$\sum_{B=1}^{B=N} \mathbf{P}_{AB} = p\, x_A \sum_{\substack{B=1 \\ B \neq A}}^{B=N} \frac{x_B \mathbf{u}_B}{\mathscr{D}_{AB}} - p\, x_A \mathbf{u}_A \sum_{\substack{B=1 \\ B \neq A}}^{B=N} \frac{x_B}{\mathscr{D}_{AB}} \tag{A4}$$

At this point we introduce the *mixed-mode diffusive flux* (see Sec. 5.3 in ref. [48]) defined by

$$\mathbf{J}_A = c\, x_A \mathbf{u}_A = c_A \mathbf{u}_A \tag{A5}$$

and use of this definition in Eq. A4 leads to

$$\sum_{B=1}^{B=N} \mathbf{P}_{AB} = (p/c)x_A \sum_{\substack{B=1\\B\neq A}}^{B=N} \frac{\mathbf{J}_B}{\mathbf{D}_{AB}} - (p/c)\mathbf{J}_A \sum_{\substack{B=1\\B\neq A}}^{B=N} \frac{x_B}{\mathbf{D}_{AB}} \tag{A6}$$

This result can be simplified if the mole fraction of species A is small enough so that

Restriction: $$x_A \sum_{\substack{B=1\\B\neq A}}^{B=N} \frac{\mathbf{J}_B}{\mathbf{D}_{AB}} << \mathbf{J}_A \sum_{\substack{B=1\\B\neq A}}^{B=N} \frac{x_B}{\mathbf{D}_{AB}} \tag{A7}$$

Since the diffusive fluxes, $\mathbf{J}_A$, $\mathbf{J}_B$, etc., tend to be the same order of magnitude, this restriction is often expressed as a constraint given by

Constraint: $$x_A << 1 \tag{A8}$$

Application of this constraint in Eq. A6 leads to

$$\sum_{B=1}^{B=N} \mathbf{P}_{AB} = -(p/c)\mathbf{J}_A \sum_{\substack{B=1\\B\neq A}}^{B=N} \frac{x_B}{\mathbf{D}_{AB}} \tag{A9}$$

and it is convenient to define a mixture diffusivity by

$$\frac{1}{\mathbf{D}_A} = \sum_{\substack{B=1\\B\neq A}}^{B=N} \frac{x_B}{\mathbf{D}_{AB}} \tag{A10}$$

so that Eq. A9 can be expressed as

$$\sum_{B=1}^{B=N} \mathbf{P}_{AB} = -(p/c\mathbf{D}_A)\mathbf{J}_A \tag{A11}$$

At this point we need to provide a precise representation of these three results for gases which is given by

$$\sum_{B=1}^{B=N} (\mathbf{P}_{AB})_{gas} = -(p_{gas}/c_{gas})\mathbf{J}_A\big|_{gas} \sum_{\substack{B=1\\B\neq A}}^{B=N} \frac{x_B}{\mathscr{D}_{AB}} \tag{A12}$$

$$\frac{1}{\mathscr{D}_A} = \sum_{\substack{B=1 \\ B \neq A}}^{B=N} \frac{x_B}{\mathscr{D}_{AB}} \tag{A13}$$

$$\sum_{B=1}^{B=N} (\mathbf{P}_{AB})_{gas} = -\left(p_{gas}/c_{gas}\mathscr{D}_A\right)\mathbf{J}_A\big|_{gas} \tag{A14}$$

For liquids we note that Eqs. A9 through A11 take the form

$$\sum_{B=1}^{B=N} (\mathbf{P}_{AB})_{liq} = -\left(p_{liq}/c_{liq}\right)\mathbf{J}_A\big|_{liq} \sum_{\substack{B=1 \\ B \neq A}}^{B=N} \frac{x_B}{D_{AB}} \tag{A15}$$

$$\frac{1}{D_A} = \sum_{\substack{B=1 \\ B \neq A}}^{B=N} \frac{x_B}{D_{AB}} \tag{A16}$$

$$\sum_{B=1}^{B=N} (\mathbf{P}_{AB})_{liq} = -\left(p_{liq}/c_{liq}D_A\right)\mathbf{J}_A\big|_{liq} \tag{A17}$$
