# Supplementary material for: Diffusion of Charged Species in Liquids
Source: Sci Rep. 2016 Nov 4;6:35211. doi: 10.1038/srep35211 (PMC5566185; doi:10.1038/srep35211)
Supplement: Supplementary Appendix B [file srep35211-s2.pdf]

Diffusion of Charged Species in Liquids

J. A. del Río[1]* and S. Whitaker[2]

1.Instituto de Energías Renovables, Universidad Nacional Autonoma de Mexico,
A.P. 34, 62580 Temixco, Mor. Mexico

2. Department of Chemical Engineering and Material Science, University of California at Davis, Davis, CA 95616, USA

* Correspondence
Dr. J.A. del Río,
Email: arp@ier.unam.mx

September 5, 2016

Appendix B: Forced Diffusion

In this appendix, we return to Eq. 50 and direct our attention to the body force that is applicable to both gases and liquids. The body force term in Eq. 50 can be manipulated to obtain

$$\rho_A(\mathbf{b}_A - \mathbf{b}) \;=\; \rho_A \mathbf{b}_A - \rho_A \sum_{B=1}^{B=N} \omega_B \mathbf{b}_B \;=\; \rho_A \mathbf{b}_A - \omega_A \sum_{B=1}^{B=N} \rho_B \mathbf{b}_B \tag{B1}$$

in which the second of Eqs. 27 has been employed. The species A body force, $\rho_A \mathbf{b}_A$, can be expressed in terms of an electrostatic force per unit mass, $\mathbf{b}_A^{(e)}$, and a gravitational force per unit mass, $\mathbf{g}$. This leads to

$$\left\{\begin{matrix}\text{body force per}\\ \text{unit volume acting}\\ \text{on species } A\end{matrix}\right\} \;=\; \rho_A \mathbf{b}_A \;=\; \rho_A \mathbf{b}_A^{(e)} + \rho_A \mathbf{g}\,, \quad A = 1, 2, \ldots, N-1 \tag{B2}$$

and use of this representation in Eq. B1 provides

$$\rho_A(\mathbf{b}_A - \mathbf{b}) \;=$$

$$= \left[\rho_A \mathbf{b}_A^{(e)} + \rho_A \mathbf{g}\right] - \omega_A \sum_{B=1}^{B=N} \left[\rho_B \mathbf{b}_B^{(e)} + \rho_B \mathbf{g}\right] \tag{B3}$$

$$= \left[\rho_A \mathbf{b}_A^{(e)} + \rho_A \mathbf{g}\right] - \omega_A \sum_{B=1}^{B=N} \rho_B \mathbf{b}_B^{(e)} - \omega_A \rho \mathbf{g}$$

Here we see that the constant gravitational force is eliminated (since $\rho_A = \omega_A \rho$) leading to the following representation for the electro-static force acting on species A:

$$\rho_A(\mathbf{b}_A - \mathbf{b}) \; = \; \rho_A \mathbf{b}_A^{(e)} - \omega_A \sum_{B=1}^{B=N} \rho_B \mathbf{b}_B^{(e)} \,, \qquad A = 1,2,...,N \tag{B4}$$

The total electro-static force acting on all species is given by

$$\left\{ \begin{matrix} \text{total electro-static force per} \\ \text{unit volume acting on the solution} \end{matrix} \right\} \; = \; \rho \, \mathbf{b}^{(e)} \; = \; \sum_{B=1}^{B=N} \rho_B \mathbf{b}_B^{(e)} \tag{B5}$$

and when the condition of electro-neutrality is valid this term can be set equal to zero. The *assumption* associated with this idea is given by

Assumption: $$\rho \, \mathbf{b}^{(e)} \; = \; 0 \tag{B6}$$

while the *restriction* [46] is stated in the form

Restriction: $$\rho_A \mathbf{b}_A^{(e)} \; >> \; \omega_A \sum_{B=1}^{B=N} \rho_B \mathbf{b}_B^{(e)} \tag{B7}$$

Deen (see page 455 in ref. [17]) indicates that electro-neutrality is often imposed on ion transport processes; however, it generally fails in the vicinity of charged surfaces (see page 782 in ref. [6], see Chapter 7 in ref. [30]). In this study we avoid charged surfaces and impose the condition of electro-neutrality so that Eq. B4 provides

$$\left\{ \begin{matrix} \text{electro-static} \\ \text{force per unit volume} \\ \text{acting on species } A \end{matrix} \right\} \; = \; \rho_A(\mathbf{b}_A - \mathbf{b}) \; = \; \rho_A \mathbf{b}_A^{(e)} , \qquad A = 1,2,...,N \tag{B8}$$

At this point we are ready to consider the species A electro-static force.

Electro-static force

Stratton (see page 96 in ref. [41]) represents the electro-static force per unit volume as

$$\mathbf{f}_e \; = \; \left\{ \begin{matrix} \text{charge per} \\ \text{unit volume} \end{matrix} \right\} \mathbf{E} \tag{B9}$$

in which $\mathbf{E}$ represents the electric field. Following Deen (see page 454, ref. [17]) among many others, we represent the *charge per mole* of species A as $z_A F$ where z_A is the valence (i.e., -2 for $SO_4^{=}$ and $+1$ for H_3O^{+}) and F is the conversion factor known as Faraday's constant. This means that the *charge per unit volume* associated with species A is

$$\begin{Bmatrix} \text{charge per} \\ \text{unit volume} \end{Bmatrix} = z_A c_A F \tag{B10}$$

and the form of Eq. B8 for species A is given by

$$\rho_A \mathbf{b}_A^{(e)} = z_A c_A F \mathbf{E} \tag{B11}$$

When the charges are in equilibrium, the electric field can be expressed as the gradient of a potential function (see page 115 in ref. [41]) and we express this idea as

$$\mathbf{E} = -\nabla\Psi \tag{B12}$$

Use of this expression in Eq. B11 gives

$$\rho_A \mathbf{b}_A^{(e)} = -z_A c_A F \nabla\Psi \tag{B13}$$

and to connect this result with the discussion in the main body of the paper we use Eq. B8 to obtain

$$\begin{Bmatrix} \text{electro-static force per unit} \\ \text{volume acting on species } A \end{Bmatrix} = \rho_A(\mathbf{b}_A - \mathbf{b}) = $$

$$= \rho_A \mathbf{b}_A^{(e)} = -z_A c_A F \nabla\Psi , \qquad A = 1,2,...,N \tag{B14}$$
