# Supplementary material for: Diffusion of Charged Species in Liquids
Source: Sci Rep. 2016 Nov 4;6:35211. doi: 10.1038/srep35211 (PMC5566185; doi:10.1038/srep35211)
Supplement: Supplementary Appendix C [file srep35211-s3.pdf]

Diffusion of Charged Species in Liquids

J. A. del Río[1]* and S. Whitaker[2]

1.Instituto de Energías Renovables, Universidad Nacional Autonoma de Mexico,
A.P. 34, 62580 Temixco, Mor. Mexico

2. Department of Chemical Engineering and Material Science, University of California at Davis, Davis, CA 95616, USA

* Correspondence
Dr. J.A. del Río,
Email: arp@ier.unam.mx

September 5, 2016

Appendix C: Alternate Analysis

Since an ideal gas mixture satisfies the equation of state given by

Ideal gas: $$p_{gas} = c_{gas} RT \tag{C1}$$

we can express Eq. 54 in the alternate form given by

Ideal gas mixture: $$\mathbf{P}_{AB}\big|_{gas} = c_{gas} RT \frac{x_A x_B}{\mathscr{D}_{AB}} \left(\mathbf{v}_B - \mathbf{v}_A\right), \quad A = 1,2,\ldots,N-1 \tag{C2}$$

This can be used as a *model* for liquid-phase diffusion to obtain

Ideal liquid mixture: $$\mathbf{P}_{AB}\big|_{liq} = c_{liq} RT \frac{x_A x_B}{\not{D}_{AB}} \left(\mathbf{v}_B - \mathbf{v}_A\right), \quad A = 1,2,\ldots,N-1 \tag{C3}$$

Here it is important to note that that $\not{D}_{AB}$ represents an empirical coefficient to be determined by experiment, and it is crucial to recognize that $\not{D}_{AB}$ and D_{AB} are related by

$$\not{D}_{AB} = D_{AB} \left(c_{liq} RT / p_{liq}\right) \tag{C4}$$

For a typical aqueous mixture at atmospheric pressure we have

$$\left(c_{liq} RT / p_{liq}\right) \approx 1200 \tag{C5}$$

thus one must not confuse the two diffusivities, $\not{D}_{AB}$ and D_{AB}.

We can express Eq. C2 in the form

$$\mathbf{P}_{AB}\big|_{gas} = p_{gas}\frac{x_A\,x_B}{\mathscr{D}_{AB}\left(p_{gas}/c_{gas}RT\right)}\left(\mathbf{v}_B-\mathbf{v}_A\right), \quad A = 1,2,...,N-1 \tag{C6}$$

and an analogous version of Eq. C3 is given by

$$\mathbf{P}_{AB}\big|_{liq} = p_{liq}\frac{x_A\,x_B}{\text{Đ}_{AB}\left(p_{liq}/c_{liq}RT\right)}\left(\mathbf{v}_B-\mathbf{v}_A\right), \quad A = 1,2,...,N-1 \tag{C7}$$

At this point we follow the development beginning with Eq. 56 and express Eqs. C6 and C7 as

$$\mathbf{P}_{AB} = p\frac{x_A\,x_B}{\textbf{Đ}_{AB}}\left(\mathbf{v}_B-\mathbf{v}_A\right), \quad A = 1,2,...,N-1 \tag{C8}$$

In this case the diffusivity $\textbf{Đ}_{AB}$ is given by

gas phase: $$\textbf{Đ}_{AB} = \mathscr{D}_{AB}\left(p_{gas}/c_{gas}RT\right) \tag{C9}$$

liquid phase: $$\textbf{Đ}_{AB} = \text{Đ}_{AB}\left(p_{liq}/c_{liq}RT\right) \tag{C10}$$

and we define the associated mixture diffusivity according to

$$\frac{1}{\textbf{Đ}_A} = \sum_{\substack{B=1\\B\neq A}}^{B=N}\frac{x_B}{\textbf{Đ}_{AB}} \tag{C11}$$

Here we follow Eqs. A1 through A11 to obtain

$$\sum_{B=1}^{B=N}\mathbf{P}_{AB} = -\left(p/c\,\textbf{Đ}_A\right)\mathbf{J}_A\,, \qquad x_A \ll 1 \tag{C12}$$

and application of Eq. C8 leads to

$$\sum_{B=1}^{B=N}\frac{x_A\,x_B}{\textbf{Đ}_{AB}}\left(\mathbf{v}_B-\mathbf{v}_A\right) = -\left(\mathbf{J}_A/c\,\textbf{Đ}_A\right), \qquad x_A \ll 1 \tag{C13}$$

We now repeat the analysis from Eq. 60 to Eq. 67 to obtain

$$\mathbf{J}_A = \underbrace{-\,\textbf{Đ}_A\nabla c_A}_{\textit{Fickian diffusion}} - \left(cRT/p\right)\underbrace{\textbf{Đ}_A\,z_A\,c_A\left(F/RT\right)\nabla\Psi}_{\textit{Nernst-Planck diffusion}} \tag{C14}$$

For an *ideal gas* we make use of Eqs. C9 and C11 leading to

ideal gas: $$\frac{1}{\textbf{Đ}_A} = \left(c_{gas}RT/p_{gas}\right)\sum_{\substack{B=1\\B\neq A}}^{B=N}\frac{x_B}{\mathscr{D}_{AB}} = \left(c_{gas}RT/p_{gas}\right)\frac{1}{\mathscr{D}_A} \tag{C15}$$

and for an *ideal liquid* Eqs. C9b and C10 provide

$$\text{ideal liquid:} \qquad \frac{1}{\text{Đ}_A} = \left(c_{liq}RT/p_{liq}\right)\sum_{\substack{B=1\\B\neq A}}^{B=N}\frac{x_B}{\text{Đ}_{AB}} = \left(c_{liq}RT/p_{liq}\right)\frac{1}{\text{Đ}_A} \tag{C15}$$

For an ideal gas we have the result given by

$$cRT/p = \left(c_{gas}RT/p_{gas}\right) = 1 \tag{C16}$$

and the use of this result along with Eq. C14 in Eq. C13 leads to

$$\text{gas phase:} \qquad \mathbf{J}_A\big|_{gas} = \underbrace{-\mathcal{D}_A\nabla(c_A)_{gas}}_{Fickian\ diffusion} - \underbrace{\mathcal{D}_A\, z_A (c_A)_{gas}\left(F/RT\right)\nabla\Psi}_{Nernst\text{-}Planck\ diffusion} \tag{C17}$$

For an ideal liquid we make use of Eq. C15 in Eq. C13 to obtain

$$\text{liquid phase:} \qquad \mathbf{J}_A\big|_{liq} = -\left(p_{liq}/c_{liq}RT\right)\text{Đ}_A\nabla c_A - \text{Đ}_A\, z_A\, c_A\left(F/RT\right)\nabla\Psi \tag{C18}$$

One can force the first term on the right hand side into the classic form for Fickian diffusion by use of the definition

$$\left(p_{liq}/c_{liq}RT\right)\text{Đ}_A = \text{Đ}_A^* \tag{C19}$$

however, this leads to the non-classical result given by

$$\mathbf{J}_A\big|_{liq} = -\text{Đ}_A^*\nabla c_A - \left(c_{liq}RT/p_{liq}\right)\text{Đ}_A^*\, z_A\, c_A\left(F/RT\right)\nabla\Psi \tag{C20}$$

It is important to keep in mind that this result is quite correct. The algebraic manipulations given here are simply that: *algebraic manipulations*. The key idea is that the classic form of Nernst-Planck diffusion given by Eq. C17 does not apply to liquids.

To complete this development, we note that one can use Eqs. C4, C10, C11, C16, C20 and A16 to show that $\text{Đ}_A^* = D_A$. This means that Eq. C20 takes the form

$$\mathbf{J}_A\big|_{liq} = \underbrace{-D_A\nabla c_A}_{Fickian\ diffusion} - \left(c_{liq}RT/p_{liq}\right)\underbrace{D_A\, z_A\, c_A\left(F/RT\right)\nabla\Psi}_{Nernst\text{-}Planck\ diffusion} \tag{C21}$$

that is identical to Eq. 70.
